# Primary health care workers perspective towards cancer in Fiji: a qualitative study

**DOI:** 10.1017/S1463423621000888

**Published:** 2022-01-13

**Authors:** Kaushal Kumar, Masoud Mohammadnezhad

**Affiliations:** School of Public Health and Primary Care, Fiji National University, Suva, Fiji

**Keywords:** barriers, cancer, Fiji, knowledge, perceptions, primary health care workers, training

## Abstract

**Background:**

By 2040, the predicted global cancer burden is expected to be more than 27 million new cancer cases per year. Understanding primary health care workers’ (HCWs) perception on cancer can highlight new ways in which cancer advocacy can be increased. This study aimed to explore the perceptions of primary HCWs in Lautoka, Fiji, towards common cancers with focus on knowledge, risk perceptions, barriers and preventive approaches.

**Methods:**

The study used a qualitative method approach. The study was conducted among primary HCWs at four purposively selected health centres in Lautoka Subdivision, Fiji, from 1 March 2021 to 1 April 2021. Focus group discussions (FGDs) were conducted with primary HCWs. A semi-structured open-ended questionnaire was used to collect data, and the FGDs were audio-recorded. These audio recordings were transcribed and analysed using thematic analysis.

**Results:**

The responses from the four FGDs with six primary HCWs in each group emerged four major themes. These themes were cancer knowledge, health professional training, barriers and challenges and awareness strategies. Primary HCWs were not fully aware about common cancers and were not confident to discuss about cancer with their patients which is an important role of primary HCWs in cancer management. This lack of knowledge was attributed to less training received in primary care setting. Barriers to accessing cancer screening included misconceptions about cancer, negative attitudes from patients, stigmatization, lack of resources at health facility and less informed health staff. Community outreach programmes, opportunistic screening, community HCWs and the concept of a cancer hub centre were awareness strategies highlighted by primary HCWs.

**Conclusions:**

Lack of knowledge about common cancers among primary HCWs is a concern that is depicted well in this study. This low knowledge was attributed to lack of training on cancers received by primary HCWs. Guidelines on cancer screening and diagnosis can be developed by the health ministry to assist primary HCWs in detecting patients at pre-cancerous stage.

## Introduction

Cancer is one of the main concerning non-communicable diseases (NCDs) around the world. In 2020, the International Agency for Research on Cancer (IARC) (2020) a branch of World Health Organization (WHO) reports that global cancer burden has increased to 19.3 million new cases and 10 million cancer deaths worldwide. By 2040, the predicted global cancer burden is expected to be more than 27 million new cancer cases per year which is almost a 50% increase in the estimated cancer cases in 2018, with majority of the cases seen in countries with low or medium Human Development Index (HDI) (Green Facts, 2020; The Cancer Atlas, 2020). Liver, colorectal, lung, stomach and prostate cancer are the most common types of cancer in men, while lung, thyroid, cervical, breast and colorectal cancer are the most common among women (World Health Organization, 2011; World Cancer Research Fund, 2019; Who.int, 2020).

Cancer risk factors are usually divided into categories such as internal factors (age, sex, inherited genetic defects), lifestyle-related factors, occupational exposures (chemicals, radioactive materials) and environmental exposures (UV radiation) (National Cancer Institute, 2015; MedicineNet, 2018; American Cancer Society, 2020; Centers for Disease Control and Prevention, 2020a). Cancer can be prevented by avoiding such cancerous agents and also by early screening (Centers for Disease Control and Prevention, 2020b).

It is estimated that over the coming decades, the low- and middle-income countries (LMICs) will hit the hardest with the growing cancer burden (Green Facts, 2020). Furthermore, in developed countries where health systems are stronger, survival rates of many types of cancer are improving due to early detection, timely diagnosis and treatment (The National Academies Press, 2016; Prager *et al.,* 2018; Who.int, 2020). It is very important for primary health care workers (HCWs) to understand patient perspectives to better diagnose and treat cancers. There may be significant differences between a HCWs’ perspective and a patient perspective in determining success of a given health care intervention (Asadi-Lari *et al.,* 2004). Hence, effective communication between a HCWs and the patient is necessary to achieve patient satisfaction, as patients who are satisfied are more likely to adhere to treatment, take an active role in health care, continue to trust the medical services and stay within a health care provider (Donabedian, 1988; Guldvog, 1999; Asadi-Lari *et al.,* 2004).

Primary HCWs are well positioned to provide detailed information and cancer care to individuals because of their accessibility in the community and their relationships with patients, in particular, their knowledge about patients’ family history, personal history, social circumstances and co-morbidities (Easley *et al.,* 2017). All HCWs around the world work tirelessly to care for their patients in an increasingly complex, inefficient and stressful environment. However, the culture and structure of the system in which they work are often poorly aligned to support their efforts to respond to patients’ needs (Committee on the Learning Health Care System in America *et al.,* 2013). Primary HCWs also face some difficulties in providing cancer information to patients (Lawrence *et al.*, 2016; Mohanty *et al.,* 2019). These difficulties include lack of information about cancers, limited knowledge, lack of guidelines, lack of time and lack of patient trust (Committee on the Learning Health Care System in America *et al.,* 2013; Lawrence *et al.*, 2016; Easley *et al.,* 2017; Mohanty *et al.,* 2019). However, primary HCWs must continue to change perceptions, educate and inform individuals about cancers.

Fiji is a small pacific island nation with a total population of 904 997 (Fiji Population, & Demographics, Maps, Graphs, 2021). NCDs are the number one killer in Fiji with majority deaths from diabetes, hypertension and cardiovascular diseases (Fiji Broadcasting Corporation, 2020; International Agency for Research on Cancer, n.d.). In Fiji, there were 260 new cancer cases recorded as of May 2020 of which 24 were recorded in May alone. In 2019, 250 cases were recorded for the whole year, out of which 29 patients have lost their lives (Fiji Broadcasting Corporation, 2020). The number of new cases is drastically increasing in Fiji, and unfortunately, cancer is the 3rd most common cause of death in Fiji. The risk of developing cancer before the age of 75 years in Fiji is 16.7%, while the risk of death from cancer before the age of 75 years is 10.2% (International Agency for Research on Cancer, n.d.).

A gap of knowledge exists since currently nil studies could be found that explores the cancer perceptions of primary HCWs. Hence, this study aimed to explore the perceptions of primary HCWs in Fiji towards common cancers with focus on knowledge, risk perceptions, barriers and preventive approaches. This study will attempt to bridge the gap between the findings from western studies and the local context, as well as attempt to plan targeted interventions to increase knowledge about cancers among the primary HCWs which will directly help increase cancer knowledge among the general public.

## Methodology

### Study design and setting

This study applied a qualitative approach using focus group discussions (FGDs) to explore the perceptions of primary HCWs towards common cancers in Lautoka Subdivision, Fiji from 1 March 2021 to 1 April 2021. A FGD is a good way to gather people from similar backgrounds with diverse characteristics such as age, gender, designation and years of health care experience to discuss a specific topic of interest and is very useful in generating a rich understanding of participants’ experiences and beliefs (Mishra, 2016). The study was conducted at the four purposively selected health centres in Lautoka Subdivision, namely Punjas Health Centre, Kamikamica Health Centre, Natabua Health Centre and Veiseisei Health Centre. These health centres are the busiest health centres in Lautoka Subdivision which cater for approximately 3000 patients per week and run both the General Outpatient services and the Special Outpatient services (SOPD) for diabetes, hypertension, dyslipidaemia/cardiac.

### Study sample

The study focused on all the primary HCWs based at the four health centres in Lautoka Subdivision within the study period. The following inclusion criteria were used: the primary HCW should be a nurse or a primary care physician based full time at the 4 health centres, should have served as a primary HCW for at least 6 months, age more than or equal to 18 years and gender either males or females. The following exclusion criteria were used: primary HCW based at other health facilities in Lautoka, primary HCWs who have previously worked full time at an oncology centre and primary HCWs who are not willing to participate in FGDs.

A purposive sampling was used which included all primary HCWs based on the four health centres in the Lautoka Subdivision, and they needed to satisfy the inclusion and exclusion criteria. There were four (4) FGDs held for primary HCWs, that is one in each health facility with six (6) participants in each FGD. Each focus group comprised of three (3) primary care physicians and three (3) nurses, with equal number of males and females.

### Data collection tool

The data collection instrument used to collect data in this research was semi-structured open-ended questionnaire to guide FGDs. The questionnaire was developed from literature review in line with the research questions of this study which has 2 sections with 13 questions in total. The first section which is the demographics had 6 questions followed by 8 open-ended questions in the second section. The FGD questions are in English version.

### Study procedure

The primary HCWs in each of the four health centres were given an introduction of the study verbally. Together with this verbal introduction, information sheet was provided to all the primary HCWs in the English language (who fulfilled the inclusion and exclusion criteria). The primary HCWs who agreed to participate in the study at their own free time were then given consent forms in English.

Once the participants gave their signed consents, the consent forms were collected and kept safely while the information sheet remained with the participants. After this, the FGDs were conducted by the principal researcher with primary HCWs in a quiet room at each health centre with each discussion lasting for at least 60 min. The discussions were recorded. Four FGDs was conducted, one at each health centre, with 6 participants in each group.

### Data management and analysis

All FGD recordings were transcribed verbatim by the principal researcher. Transcription was done on the same day of the FGD. A review of transcriptions was done to correct errors and to remove references of names and places to ensure anonymity for the participants. Once the transcriptions were clarified, data analysis was carried out.

Thematic analysis was used to analyse the data in this study. Thematic analysis is a qualitative research method for identifying, analysing, organizing, describing and reporting themes found within a data set (Vaismoradi *et al.,* 2016; Vaismoradi & Snelgrove, 2019). The principal researcher and the principal supervisor read and re-read all FGD transcripts and identified similar phrases and words for which numbers were assigned. Different phrases and words were also identified to analyse the data further. The coded data that had similar characteristics were grouped together. Once grouping of similar data was completed, descriptive themes and subthemes were identified to reflect the perceptions of participants (Vaismoradi & Snelgrove, 2019). The themes and subthemes were checked by the principal supervisor as well.

### Study rigour

Four criteria were identified that contributed to study rigour. These criteria consisted of credibility, transferability, dependability and confirmability. Some ways to make the study more rigour included the following: a short introduction of the study provided verbally by the researcher to all primary HCWs, flyers that contained information about the research placed at each health centre 2 weeks prior to data collection, FGDs were conducted over the period of 1 month and each FGD lasted for at least 60 min, all discussions were recorded, principal supervisor checked each step of the research, review of transcriptions were done to correct errors and purposive sampling technique.

### Ethical considerations

Ethical approval for this study was obtained from Fiji National University’s (FNU) College Health Research Ethics Committee (CHREC) and Fiji National Research Ethics Review Committee (FNRERC). Written informed consent (with rights to withdraw without any consequences) was taken from primary HCWs, and assurance of confidentiality and anonymity was provided to them throughout the course of the study and afterwards as well. The primary HCWs were advised that participation in this study is voluntary, and they can leave the study at any time without any consequences.

## Results

### Characteristics of the participants

Table 1 presents the characteristics of the participants of FGD among primary HCWs. There were four FGDs conducted in this research, one in each health facility with a total of 6 participants in each FGD. Twenty-four primary HCWs (12 females and 12 males) agreed to be part of the FGD. Of these 24 participants, the majority were of the Indo-Fijian ethnicity (75%). There were twelve nurses and twelve medical officers who took part in the FGD. In terms of years of working experience at primary health care setting, majority participants have been working for 3 years at the health facility (41.7%). Each participant was assigned a number and coded from participant 1 to 24. The FGD to which participants belonged to is also indicated in the quotations references.

**Table 1.** Characteristics of study participants (*n* = 24)

Characteristics		Frequency	Percentage
Gender	Male	12	50
	Female	12	50
Ethnicity	iTaukei	6	25
	Indo-Fijian	18	75
Designation of primary ^HCWs^	Medical Officers	12	50
	Nurses	12	50
Years of work experience	≤ 2 years	7	29.2
	3–4 years	15	62.5
	≥ 5 years	2	8.3

HCWs = health care workers.

### Themes and subthemes

After completion of thematic analysis, four major themes emerged, this included the following: cancer knowledge, health professional training, barriers and challenges and awareness strategies. Under these, major themes and subthemes were identified, as summarized in Table 2. In this section, the quotes will be based on the number of FGD and primary HCWs age, gender and position (SN for Staff Nurse and MO for Medical Officer), for example, FG1, a 35-year-old male SN, that means a Staff Nurse participant from FGD 1.

**Table 2.** Themes and subthemes of FGD

Themes	Subthemes
Cancer knowledge	Risk factors
	Diagnostic modalities
	Cancer management
	Cancer discussion preparedness
Health Professional training	University-level training
	Primary care training
Barriers and challenges	Worry and Fear
	Stigmatization
	Resources and communication
Awareness strategies	Breaking barriers
	Community outreach programmes

FGD= Focus Group Discussion

### Theme 1: cancer knowledge

The first few questions posed to the primary HCWs were about their knowledge on cancer risk factors, diagnosis, management, screen modalities and preparedness on cancer discussion. The responses of participants were noted under four subthemes, namely risk factors, diagnostic modalities, cancer management and cancer discussion preparedness.

### Risk factors

The primary HCWs felt that they knew about common risk factors; however, they did not have much knowledge about specific risk factors for specific cancers.

‘I know about common cancers however like lung cancer, liver cancer or bowel cancer, I don’t know much about these. I guess everyone here is most versed with risk factors and screening for breast, cervical and prostate cancer. For diagnosis and treatment options, we do not have any idea about it.’ (FG2, a 36–year-old female MO)

The participants had the most information about breast and cervical cancer risk factors.

‘We are mainly aware about risk factors and screening for breast cancer and cervical cancer but not the other cancers. Usually we focus on female cancers more than male cancers.’ (FG1, a 29-year-old female MO)

Participants have stated smoking as a common risk factor for majority of the cancers.

‘I am aware about risk factors for common cancers in Fiji. Majority of the risk factors are same. Smoking is an important risk factor for almost all cancers.’ (FG1, a 40-year-old male SN)

Other common risk factors identified by participants are unhealthy lifestyle and genetics. They view these as important components to cancer identification.

‘Yes, we are aware about common cancers in Fiji namely breast cancer, cervical cancer and prostate cancer. For other cancers we really don’t know much about diagnosis and treatment. However, we are aware mainly about general risk factors for cancer such as unhealthy lifestyle and genetics or strong family history. We know bits and pieces about cancers.’ (FG3, a 27-year-old female MO)

Family history and genetics are important indicators for cancer screening.

‘We are familiar with screening and risk factors for common cancers. Genetics or positive family history is a very common risk factor for all cancers. I believe anyone with a positive family history of cancer must get tested for cancers.’ (FG4, a 28-year-old female MO)

### Diagnostic modalities

The participants stated that they are aware about diagnostic modalities for common cancers such as breast cancer and cervical cancer. However, they have less information about diagnosis for prostate, liver and colorectal cancer.

‘We are sort of familiar about screening modalities available and diagnosis specially for common cancers. Prostate cancer, liver cancer and bowel cancers are the left out ones that we hardly discuss so we are unaware about screening and diagnosis for these. Since we don’t know much about bowel and rectal cancer screening and diagnosis, we usually keep treating patients with different medications unaware that it could be cancer.’ (FG1, a 29-year-old female MO)

### Cancer management

Majority of the primary HCWs stated that they are unaware about cancer treatment options because it is out of their scope of work.

‘I am unfamiliar about treatment options for cancers. I have a bit of knowledge about breast cancer treatment because it’s one of the common cancers in Fiji but for other cancers I am not sure how it gets treated’ (FG2, a 29-year-old female MO)

Primary HCWs stated that cancer management is mostly dealt with tertiary hospitals.

‘We have general idea about breast cancer treatment but unsure about treatment options for other cancers. I guess we don’t focus much on treatment options at primary care level hence that could be the reason why we don’t know much about it.’ (FG1, a 29-year-old female MO)

### Cancer discussion preparedness

Many participants stated that they are not prepared to discuss about cancer with patients at primary care level.

‘We are not very confident with discussion of cancer with patients. Especially the counselling part for even screening for cancers. Like for example to tell someone that we want to do a Per Rectal (PR) examination to screen for cancer is difficult to explain to patients.’ (FG1, a 29-year-old female MO)

Few primary HCWs stated that the emotional stress experienced by patients after cancer discussion is difficult for them to manage.

‘To discuss with someone about cancer is like giving someone anxiety so we should be prepared to deal with this anxiety and wave of questions. Like for some patients when we tell that we want to test for cancer, they get really scared and ask a lot of questions and we don’t know how to answer it.’ (FG1, a 29-year-old female MO)

The participants felt that discussion about screening methods is easier; however, discussion about diagnosis and treatment plan for cancer is difficult since they are not well versed with the concept.

‘I am a bit comfortable to discuss about cancers with patients especially the preventative and screening part which is very easy. The problem is the diagnosis and treatment part of cancers which we are unaware about.’ (FG3, a 26-year-old female MO)

Another primary HCW stated that:

‘In terms of counselling before diagnosis, we are not very comfortable and prepared to discuss about cancer with patients and after diagnosis counselling is definitely out of our scope of work as general outpatient workers.’ (FG2, a 29-year-old female MO)

Patients who present with signs and symptoms of cancer provoke a health professional to discuss about cancer screening. As stated by one participant:

‘When patients come with signs and symptoms of cancer, then only I discuss with the patient about cancer screening and diagnosis otherwise I do not advice about screening routinely to patients.’ (FG3, a 27-year-old female MO)

### Theme 2: health professional training

The next set of questions that were asked to the participants were in regard to cancer training. The participants divided their answers in two sections that is university-level training and primary care level training.

### University-level training

All the participants received some form of training in cancer while they were pursing their undergraduate programmes.

‘During university days, we learnt about cancers from our lecturers and also we were given a chance to attach to the oncology unit. This gave us some exposure to cancer information.’ (FG1, a 28-year-old female SN)

Some primary HCWs stated that they were attached in the oncology unit during their university days.

‘In medical school year 6, when we were doing our clinical rotations, we were told to attach to oncology unit to know about certain screening, diagnosis and management modalities of cancers.’ (FG2, a 27-year-old female MO)

### Primary care level training

All participants were on the same page when discussing about training at primary care level. All primary HCWs have stated that they have not received any training on cancers at primary care level.

‘I haven’t received any training on cancer counselling in primary care. It is very important for us primary HCWs to be trained to counsel on cancer as we are the main entry point for majority patients.’ (FG4, a 26-year-old female SN)

Few participants stated that:

‘I think if my patient read an article about breast cancer before coming to me to ask about screening, she would definitely have more information about cancers then me hence there is a big need for training on cancers in primary health care level.’ (FG2, a 36-year-old female MO)

Primary health care is usually the first line of health services offered and HCWs have stated that they need to be trained first as well.

‘Patients don’t go directly to the tertiary hospital. They first are seen by primary HCWs and if we don’t know much about cancers ourselves we might send a probable cancer patient home saying you don’t require screening.’ (FG4, a 36-year-old female SN)

Few participants stated that cancer is a neglected topic in most workshops attended.

‘None of us have received any formal training on cancer counselling in primary care setting. In the PEN model training for NCD, cancers is one of the component but the course convenors neglected cancer and said let’s focus on only diabetes, hypertension and cardiovascular diseases.’ (FG1, a 29-year-old female MO)

There is a need for training on pre-test and post-test counselling for cancer. This will enable the primary health care to be more confident.

‘I think it’s something like HIV/AIDs, like we have to do pre-test counselling and post-test counselling and for that definitely we need training. Cancer is really something we need to focus on in terms of counselling.’ (FG1, a 40-year-old male SN)

Few participants stated that as health professionals, they need to know how to remove stigma associated with cancer.

‘There is a huge stigma associated with the word cancer and as soon as a patient comes in saying he thinks he has cancer and would like to get screened, we as health professionals need to have very accurate information and plus we need know how to deal with such patients to effectively manage them.’ (FG1, a 29-year-old female MO)

One participant added that the concept of cancer guidelines would be very useful.

‘Even not a formal 2 or 3 day workshop, a simple flowchart kind of booklet similar to the IMCI guidelines would help us detect and manage cancer easily.’ (FG4, a 36-year-old female SN)

### Theme 3: barriers and challenges

From the FGD on barriers and challenges, few subthemes emerged such as worry and fear, stigmatization, resources and communication.

### Worry and fear

Many participants stated that patients avoid cancer screening due to misconceptions about cancer.

‘One of the barrier is the misconception among public about cancer. For example I went for a outreach visit and asked the audience as to why they don’t come to health centre for screening and they have this idea that when we go for pap smear for cervical cancer screening, it means we have cervical cancer already. Most of them are afraid to come to health centre for cancer screening because as soon as they come they think they already have cancer.’ (FG1, a 40-year-old male SN)

Various myths surrounding cancer also stops patients from getting screened. One of the primary HCW stated that:

‘Like last week during an outreach one of the village participants told me that as soon as they have sex with their partner, they should wash their virginal area to wash away the semen. If the semen stays there, it can cause cancer. So she was very confident that she will not get cancer and will not come for screening because she does this routine after sexual intercourse.’ (FG1, a 26-year-old male SN)

Another participant stated that:

‘I guess the main barrier is lack of information about cancers. Frequently when patients talk about cancer that means death to them. There is a lot of myths about cancer that is a hindrance to cancer screening as well.’ (FG3, a 37-year-old male SN)

Together with myths and fear of cancers, many patients tend to use herbal medicine to treat themselves and hence present very late to the health facility.

‘Fear is an important barrier to screening. Nobody wants to know if they cancer or not. Traditional medicine is another hindrance to screening. Some patients would stay with a huge fungating cancer mass but choose to go for herbal medicine.’ (FG2, a 36-year-old female MO)

### Stigmatization

Participants stated that stigmatization is a huge barrier to cancer screening. Most people do not turn up for cancer screening since they are worried as to what other people might think about them.

‘Especially in a village setting, they don’t come to the nearby health centre because people will start talking about I and cause stigmatization.’ (FG1, a 29-year-old female MO)

One participant stated that negative attitude from the patients is also a barrier to cancer screening.

‘Some people don’t want to come because they have this negative attitude towards cancer. To them, cancer is death so why get screened and get worried that you have cancer and die earlier with that stress in your mind.’ (FG2, a 56-year-old female SN)

### Resources and communication

The participants have highlighted that they are aware about the screening for common cancer but they do not advise their patients on a daily basis for screening because in most instances these screening tools are unavailable due to resource constrains.

‘Recently I haven’t been advising any patients about pap smear for cervical cancer screening because most patients have told me that the pap smear is not available at government facilities. So being resource restrained is one of the barriers to discuss to patients about cancer.’ (FG2, a 36-year-old female MO)

Few participants highlighted that these screening facilities are difficult to access by patients.

‘The screening facilities are a bit difficult to access because majority patients are unaware where the tests are being conducted or simply they don’t know where to go while some are aware, they still do not present because of previous experience of lack of supplies for screening.’ (FG4, a 29-year-old female SN)

Language barriers are also evident in our society. When patients are explained about cancer in their own language, they understand better.

‘Language is another barrier. We have to make patients understand in the languages they speak to better combat the issue of cancer.’ (FG3, a 30-year-old male MO)

Some participants have stated that the long cancer follow-up timeframe is an issue hindering cancer screening accessibility.

‘Booking of clinics to tertiary hospital is also an issue. When we screen someone for cancer, firstly the results are late and patients are lost to follow-up sometimes. Secondly, if results come quickly, the referral to tertiary level clinics is difficult since they get long clinics dates and by this time we have lost the patient again. A separate priority line for cancer detection and diagnosis at the tertiary hospital would be a great help getting more people to get screened. Like sort of a oncology centre where u can discuss directly about cancer signs and symptoms and they get a earlier date at the tertiary hospital to be diagnosed.’ (FG1, a 40–year-old male SN)

Few primary HCWs stated that:

‘The whole process of cancer detection, diagnosis and treatment is a long process and people don’t want to spend that much time on cancers hence they don’t present early to health facilities. This delay in diagnosis is a huge discouragement for patients.’ (FG3, a 26-year-old female MO)

As stated by one primary HCW, lack of cancer knowledge among medical staff can also be a barrier for screening.

‘Some people walk into health centre to ask for cancer screening but they get discouraged when the medical staffs are unaware about cancer screening in detail so they choose not to get screened.’ (FG3, a 27-year-old female MO)

Some participants stated that screening for cancer is only advised when they find signs and symptoms of cancer so in this way some probable cancers can be missed.

‘We only screen for cancers if a patient presents with symptoms such as for men when they have urinary problems then only we screen for prostate cancer.’ (FG4, a 28-year-old female MO)

Majority primary HCWs stated that they work in a busy environment, and they tend to forget discussing with patients about cancer screening.

‘We hardly advice our patients about cancer screening because the facility that we are working in is very busy hence there is less time to routinely discuss about cancer screening therefore we only screen symptomatic patients.’ (FG2, a 26-year-old male SN)

### Theme 4: awareness strategies

Further follow-up questions asked to the participants were regarding cancer awareness strategies. Subthemes highlighted here were breaking barriers, community outreach programmes and community HCWs.

### Breaking barriers

One of the main ways to create awareness about cancers is to firstly break all barriers surrounding cancer screening.

‘I guess we should incorporate cancer screening services in the health centre setting to screen routinely for cancers and this way we would reduce stigmatization and other transportation barriers etc.’ (FG4, a 26-year-old female SN)

Participants also discussed the idea of having a specialized cancer clinic to deal with screening, diagnosis and treatment of cancers.

‘An important concept would be if there is a specialized centre to deal with common cancer screening and diagnosis similar to a diabetic hub centre which looks into all aspects of diabetes exclusively.’ (FG2, a 29-year-old female MO)

Few of the participants also stated about the negative side of developing a specialized clinic for cancer.

‘The cancer hub idea is good but then we might increase stigmatization for patients like for example when we first started a STI hub centre people didn’t come because the thought was that everyone who went there had a sexually transmitted infection.’ (FG2, a 56–year-old female SN)

### Community outreach programmes

Community outreach programmes are tested and successful strategies for public health awareness. Many participants stated that there should be an increase in number of community outreaches and to include one to one session to combat the growing issue of cancer.

‘Awareness session in community is a useful strategy. However first we need to have a whole group session and then one to one session because people usually tend to be ashamed to speak in public so they would be more confident to speak face to face with a health care personnel.’ (FG1, a 40–year-old male SN)

One to one sessions are very effective as stated by few participants.

‘Some ethnicities have taboos so they are more comfortable to discuss cancer issues with medical professionals in a one to one discussion.’ (FG3, a 48-year-old female SN)

One participant stated that:

‘Going for village outreach is an important way to relay information about cancer to patients. When we give public speeches about cancers people hardly listen but when we go house to house and engage people in a discussion about cancer, I think people will come up to get screened and all doubts about cancer can be cleared easily.’ (FG4, a 28–year-old female MO)

Community outreach programmes are good but in order to facilitate it, more staffing is required and more training for medical staffs.

‘Awareness campaigns in communities is good but we always face staffing shortages hence less community outreaches are done. The other important aspect is that these staffs going for outreaches should be well trained to rely accurate information to the public.’ (FG1, a 29-year-old female MO)

Another participant stated that mass media plays a huge role in awareness.

‘In this current world everyone has access to a smart phone so I believe other avenues such as social media and mass media can be used to relay information about cancers.’ (FG3, a 37-year-old male SN)

## Discussion

This study aimed to explore the perceptions of primary HCWs in Lautoka, Fiji, towards common cancers with focus on knowledge, risk perceptions, barriers and preventive approaches. It was found from this study that primary HCWs were not fully aware about common cancers and cancer risk factors. Primary HCWs were not confident to discuss about cancer to their patients and this was attributed to less training received by primary HCWs on cancer. Primary HCWs stated many difficulties patients face to access cancer information and screening which include misconceptions about cancer, cancer myths, negative attitudes from patients, stigmatization, language barriers, lack of resources at health facility, less informed health staff and longer waiting times.

### Cancer knowledge

The primary HCWs seemed to have some knowledge about common cancer in Fiji but not very detailed information. This is of particular concern as primary HCWs should be well versed with all common cancers and their diagnosis and management in order to better educate the public. This low knowledge about cancers could be attributed to the lack of in-service training received by primary HCWs. The primary HCWs were aware of all common cancers briefly; however, they focused much on breast cancer, cervical cancer and prostate cancer. The primary HCWs stated that these are the three cancers they commonly encounter in the General Outpatient Department (GOPD) setting, and majority articles or guidelines are based on these three cancers in Fiji. Similarly, a study highlighted that health workers had good knowledge of important risk factors; however, over two-fifth did not know that high parity was a risk factor of cervical cancer (Umuago *et al.,* 2020). Another study highlighted that 30.2% of midwives and nurses had full knowledge, 42.9% had missing knowledge, and the remainder of them did not know about breast cancer at all (Bulut, 2017).

The primary HCWs stated that they were well versed with risk factors for cancer; however, they had less information about diagnostic modalities and cancer management. Few participants stated that they knew about diagnostic modalities for common cancers such as breast and cervical cancer since these are the most commonly seen cancers in the GOPD setting. Majority of the participants highlighted that they had very less information about cancer management. This finding is evident as cancer management is dealt by tertiary level consultants and require expertise in the area of oncology.

### Health professional training

It was found in this study that primary HCWs are not prepared to discuss cancer in detail with patients. Primary HCWs stated that they would be able to discuss about cancer risk factors and screening very easily for common cancers; however, diagnostic tests and management of cancers are difficult to discuss since they are not aware about this area themselves. One study highlighted that continuous training of primary care workers, extending the screening programmes and regulating health policies all positively impacted health. Because of the role of primary HCWs in preventative health services, we should determine the knowledge levels of health care personnel through epidemiological studies and take actions to organize in – service training for them (Institute of Medicine (US) Committee on Assuring the Health of the Pacific in the 21st Century, 2002; Can *et al.,* 2014).

This low knowledge about all cancers and lack of confidence in cancer discussion can be attributed to lack of cancer training in primary health care setting (Committee on Improving the Quality of Cancer Care *et al.,* 2013). Primary HCWs stated that the only cancer training they received was in university level during their undergraduate programmes; however, they have not received any training while working in the primary health care sector. Another factor highlighted by primary HCWs is that the topic of cancer is mostly neglected during training in primary health care level. These findings provided useful insight that can be used to guide training in primary health care setting. Primary HCWs stated that they need training on pre-test and post-test counselling for cancer in particular. A set of protocols or guidelines on cancer would be very beneficial to assist primary HCWs (Woolf *et al.,* 1999; Klein, 2002; Wang *et al.,* 2018).

### Barriers and challenges

Primary HCWs stated that they were aware about diagnostic modalities for breast cancer and cervical cancer, however unaware about other cancers. It was concerning to note that primary HCWs did not have all information about cancer screening as they are the ones who give advice to the general public and they should be well versed with each element of cancer. This finding was similar to literature as it was found that primary HCWs had poor knowledge and skills about cervical cancer in Nigeria (Onyenwenyi & Mchunu, 2019).

Primary HCWs stated many difficulties patients face to access cancer information and screening. These barriers include misconceptions about cancer, cancer myths, negative attitudes from patients, stigmatization, language barriers, lack of resources at health facility, less informed health staff and longer waiting times. Fiji being a small island nation with rich cultural background, it is very common for patients to feel stigmatized when discussing about cancer. Many patients would think cancer means death. This finding is consistent with literature as one study found that half of the participants had inadequate knowledge of cancer screening methods (50.3% for Pap smear, 57.5% for mammography, 68.4% for colonoscopy, and 54.3% for faecal occult blood) (Soylar *et al.,* 2020). The participants highlighted worry, fear and stigmatization as key barriers to cancer screening. Factors such as high cost, too busy and inadequate distribution of clinics were cited as barriers to breast cancer early detection (Lamyian *et al.,* 2007; Akuoko *et al.,* 2017; Pittalis *et al.,* 2020). The reason for not performing BSE was declared as ‘Do not know how to perform’ (Yavan *et al.,* 2010; Bansode, 2020). This poor knowledge about cancer screening can be attributed to poor cancer awareness among the general public. Effective health communication has been found to be a very important motivation factor to screening behaviour (Bener *et al.*, 2002; Bernhardt, 2004; Vermeir *et al*., 2015).

Furthermore, participants stated that they are only prompted to screen individuals when they are symptomatic. Participants from a study conducted by Tatari *et al.* (2020) believed that cancer screening was only required if women has symptoms. Lack of resources to actively screen for cancer was another barrier highlighted by the primary HCWs. Primary HCWs also stated that the low knowledge level among the health professionals is also a barrier for screening. When patients present to health facility they look up to the health professional for accurate and detailed information about cancer however sometimes, the primary HCW is himself/herself not well aware about cancer and this serves as a discouragement for patients seeking cancer information (Abdel *et al.,* 2020; Kanu *et al.,* 2021). Understanding these barriers from the primary HCWs point of view and collaborating with patients’ views can help health systems develop more policies or interventions to remove these barriers.

### Awareness strategies

Many awareness strategies were highlighted by the primary HCWs. The key component that primary HCWs believed was to remove barriers in order to promote more screening and better accessibility of cancer information. The participants also discussed the concept of having a cancer hub which will serve as a specialized care centre for all patients seeking cancer screening. However, the problem of stigmatization can also develop with this cancer hub development. An alternative to cancer hub is incorporating cancer screening into GOPD services at health facilities to reduce stigma. Majority participants highlighted that community outreach campaigns are the best way to create awareness. This is the best way to engage communities into decision-making about their own health and will also help remove stigma associated with cultural beliefs. When HCWs take the health care to the communities, it removes many barriers and improves cancer screening. Literature highlighted similar views that cancer awareness can be done by providing screening facilities in strategic locations, especially in rural areas and setting up health campaigns to educate and provide early exposure of cancer to everybody (Lahijanian, 2011; Samat *et al.,* 2014). Government and non-government health planners have the responsibility for the provision of public health care facilities and screening must be considered the best choice for reducing mortality (Lamyian *et al.,* 2007). HCWs need to be targeted first because of their pivotal role in any future screening programme (McCarey *et al.,* 2011).

### Limitations

Findings of this research must be interpreted within the context of its limitations. These limitations include due to cross sectional design, study is limited to primary HCWs in Lautoka and findings may not be generalized to Fiji’s population. Furthermore, study findings included only primary HCWs and it would be ideal for future researchers to include all general practitioners. Another limitation found was that this study was conducted only in health facilities in Lautoka which are mostly situated in urban areas, hence leaving out the health facilities which are situated in rural areas who might have different perceptions about cancer.

## Conclusion

In conclusion, primary HCWs’ knowledge about common cancers and cancer risk factors was moderate and not too detailed. Primary HCWs were prepared to discuss risk factors for common cancers; however, they were not too well versed with diagnosis and management of cancers. The general public usually relies on the medical staff to give them information about cancer; however in this study, the primary HCWs discussed that they were not prepared to discuss about cancer with the patients. The HCWs attributed this to lack of training in primary care setting on cancers and simply negligence of the cancer topic. Primary HCWs stated patient barriers that include misconceptions about cancer, cancer myths, negative attitudes from patients, stigmatization, language barriers, lack of resources at health facility, less informed health staff and longer waiting times. Community outreach programmes, house to house visits and specialized cancer screening centres were recommendations given to increase cancer awareness among the general public. It is also recommended that more focus is put on training for primary HCWs on cancer pre-test counselling and the health ministry must conduct quarterly workshops/training on cancer. Guidelines on cancer screening and diagnosis can be developed by the health ministry to assist primary HCWs in detecting patients at pre-cancerous stage.
